# The Role of the Membrane-Associated Domain of the Export Apparatus Protein, EscV (SctV), in the Activity of the Type III Secretion System

**DOI:** 10.3389/fmicb.2021.719469

**Published:** 2021-08-03

**Authors:** Boško Mitrović, Shir Lezerovich, Neta Sal-Man

**Affiliations:** The Shraga Segal Department of Microbiology, Immunology and Genetics, Faculty of Health Sciences, Ben-Gurion University of the Negev, Beersheba, Israel

**Keywords:** type III secretion system, export apparatus, SctV, transmembrane domain, oligomerization

## Abstract

Diarrheal diseases remain a major public health concern worldwide. Many of the causative bacterial pathogens that cause these diseases have a specialized protein complex, the type III secretion system (T3SS), which delivers effector proteins directly into host cells. These effectors manipulate host cell processes for the benefit of the infecting bacteria. The T3SS structure resembles a syringe anchored within the bacterial membrane, projecting toward the host cell membrane. The entry port of the T3SS substrates, called the export apparatus, is formed by five integral membrane proteins. Among the export apparatus proteins, EscV is the largest, and as it forms a nonamer, it constitutes the largest portion of the export apparatus complex. While there are considerable data on the soluble cytoplasmic domain of EscV, our knowledge of its membrane-associated section and its transmembrane domains (TMDs) is still very limited. In this study, using an isolated genetic reporter system, we found that TMD5 and TMD6 of EscV mediate strong self-oligomerization. Substituting these TMDs within the full-length protein with a random hydrophobic sequence resulted in a complete loss of function of the T3SS, further suggesting that the EscV TMD5 and TMD6 sequences have a functional role in addition to their structural role as membrane anchors. As we observed only mild reduction in the ability of the TMD-exchanged variants to integrate into the full or intermediate T3SS complexes, we concluded that EscV TMD5 and TMD6 are not crucial for the global assembly or stability of the T3SS complex but are rather involved in promoting the necessary TMD–TMD interactions within the complex and the overall TMD orientation to allow channel opening for the entry of T3SS substrates.

## Introduction

Diarrheal diseases are a major global health concern and are considered the second leading cause of death in children under the age of five. According to the World Health Organization (WHO), there are nearly 1.7 billion cases of childhood diarrheal disease per year with an estimated 500,000 deaths annually. One of the main infectious agents of pediatric diarrhea is enteropathogenic *Escherichia coli* (EPEC; Clarke et al., 2002). This pathogen was related to a series of outbreaks of infantile diarrhea in the 1940s and 1950s (Robins-Browne, 1987). While EPEC is no longer considered to be an important cause of acute diarrhea in many countries, there has been a recent reemergence with severe disease outcomes being associated with EPEC infections (Croxen et al., 2013).

Enteropathogenic *E. coli* belongs to a family of bacteria that form a distinctive histological lesion in the intestinal epithelium, collectively called attaching and effacing (A/E) pathogens (Goosney et al., 2000). In the A/E lesion, the bacteria tightly attach to the host’s intestinal epithelial cells, causing a disruption of the brush border microvilli and promoting formation of actin pedestals that elevate the pathogen above the epithelial cell. This morphology is mediated by a protein transport nanomachine termed the type III secretion system (T3SS; Buttner, 2012; Deng et al., 2017; Wagner et al., 2018). The T3SS delivers virulence factors directly into host cells, and these manipulate the host cell cytoplasm rearrangement. The injected effectors also interfere with and modify critical cellular pathways to improve bacterial survival and replication (Bhavsar et al., 2007). The core architecture of the T3SS consists of a basal body embedded within the bacterial membranes, a periplasmic inner rod, a transmembrane export apparatus, and a cytosolic platform, which includes an ATPase complex and the C-ring. In addition, a distinct hollow needle is assembled on the extracellular face of the basal body, which is linked in A/E pathogens to an extracellular long filament, and a pore complex at the host membrane to create a channel for protein secretion (Buttner, 2012).

The T3SS structural genes are encoded within the bacterial chromosome on a large 35-kbp genomic pathogenicity island called the locus of enterocyte effacement (LEE). The LEE is organized into seven operons (LEE1–LEE7) that encode structural proteins, as well as regulators and several protein effectors (Elliott et al., 2000; Deng et al., 2004; Franzin and Sircili, 2015; Gaytan et al., 2016). The export apparatus, which is found at the center of the inner membrane ring and facing the cytoplasmic side, is among the most conserved substructures within the T3SS complex. This structure is essential for secretion and acts as the entry portal for the T3SS substrates. The export apparatus is assembled from five highly conserved membrane proteins, named EscR, EscS, EscT, EscU, and EscV, which were shown to form a multimeric protein complex with a stoichiometry of 5:1:4:1:9, respectively, in the homologous T3SS of *Salmonella typhimurium* (Kuhlen et al., 2018). The complexity of this structure is illustrated by the estimation that a total of 104 transmembrane domains (TMDs) are involved in its formation (Zilkenat et al., 2016). Among the export apparatus components, EscV, which is named SctV according to the T3SS unified nomenclature (Wagner and Diepold, 2020), is the largest protein (72 kDa), and because it forms a nonamer, it constitutes the largest portion of the export apparatus complex.

EscV is divided into two large domains: an N-terminal region with seven to eight predicted TMDs and a C-terminal cytoplasmic domain (Wagner et al., 2010; Abrusci et al., 2013). The presence of a putative N-terminal cleavable signal sequence suggests that EscV is directed to the inner membrane through the *sec* pathway (Garmendia et al., 2005), and it was found that its membrane localization was independent of the T3SS (Gauthier et al., 2003). EscV and its homologs in *Salmonella* and *Shigella* (InvA and MxiA, respectively) were shown to oligomerize and form a cytoplasmic homo-nonameric ring that is located directly below the secretion pore and above the ATPase complex (Abrusci et al., 2013; Bergeron et al., 2013; Majewski et al., 2020).

EscV and its homologs in both the virulent and flagellar T3SSs have been implicated in the recruitment of T3SS substrates, chaperones, and proteins from the “gatekeeper” family of proteins to the T3SS apparatus as part of the regulation process of hierarchical secretion of T3SS substrates (Diepold et al., 2012; Minamino et al., 2012; Abrusci et al., 2013; Kinoshita et al., 2013; Portaliou et al., 2017). The binding between EscV and various T3SS cargo proteins was shown to occur via EscV’s cytoplasmic C-terminus (Minamino et al., 2012; Gaytan et al., 2016; Shen and Blocker, 2016; Portaliou et al., 2017). Mutations in two amino acid residues located on the surface of MxiA, the *Shigella* SctV homolog, were shown to lead to twofold to threefold increased secretion of the IpaH effector compared to the wild-type (WT) strain (Shen and Blocker, 2016).

Overall, these studies indicated that the SctV family of proteins is part of the export gate complex where it forms an IM pore, which is required for the assembly and proper function of the T3SS, and acts as a substrate selection checkpoint. Nevertheless, although the EscV is an integral membrane protein that contributes more than half of the TMDs of the export apparatus, most of the available information about this protein is related to its soluble domain. Therefore, in this study, we investigated the role of EscV TMDs in protein function and their involvement in global T3SS assembly and activity.

## Materials and Methods

### Bacterial Strains

WT EPEC O127:H6 strain E2348/69 (streptomycin-resistant strain) and EPEC-null mutants (Δ*escN* and Δ*escV*; Gauthier et al., 2003) were used to evaluate the T3SS and translocation activities (Table 1). *E. coli* BL21 strain (λDE3) and *E. coli* DH10B were used for protein expression and for plasmid handling, respectively (Table 1). *E. coli* FHK12, which encodes β-galactosidase under the control of the *ctx* promoter, and *E. coli* PD28 (a *malE-*deficient *E. coli* strain) were used for assessment of TMD oligomerization (Table 1). The strains were grown at 37°C in a Luria–Bertani (LB) broth or Dulbecco’s Modified Eagle Medium (DMEM) with or without IPTG in the presence of the appropriate antibiotics. Antibiotics were used at the following concentrations: carbenicillin (100 μg/ml), streptomycin (50 μg/ml), and chloramphenicol (30 μg/ml). Bacterial growth was recorded at 600 nm every 30 min on the Infinite 200 PRO multimode plate reader (Tecan Group Ltd., Switzerland).

**TABLE 1:** Strains and plasmids used in this study.

Strains	Description	References
WT EPEC	EPEC strain E2348/69, streptomycin resistant	Iguchi et al., 2009
EPEC Δ*escV*	Non-polar deletion of *escV*	Gauthier et al., 2003
EPEC Δ*escN*	Non-polar deletion of *escN*	Gauthier et al., 2003
*E. coli* DH10B	For plasmid handling	Durfee et al., 2008
*E. coli* FHK12	*E. coli* strain in which the *ctx* promoter is fused to a *lacZ* gene	Kolmar et al., 1995
*E. coli* PD28	A *malE*-deficient *E. coli* strain	Duplay and Szmelcman, 1987
*E. coli* BL21 (λDE3)	For protein expression	Promega
**Plasmids**		
pEscV_wt_ (pACYC184)	Untagged EscV in pACYC184	Gauthier et al., 2003
pEscV_wt_-V5 (pACYC184)	V5 C-terminal tagged EscV in pACYC184	This study
pEscV_wt_-HSV (pACYC184)	HSV C-terminal tagged EscV in pACYC184	Gauthier et al., 2003
pEscV_wt_-V5 (pSA10)	V5 C-terminal tagged EscV in pSA10	This study
pEscV_wt_-HA (pSA10)	HA C-terminal tagged EscV in pSA10	This study
pEscV_wt_-His (pSA10)	His C-terminal tagged EscV in pSA10	This study
pEscV-TMD5_ex_-His (pSA10)	C-terminal tagged EscV with an 7L9A sequence instead of the original TMD5 in pSA10	This study
pEscV-TMD6_ex_-His (pSA10)	C-terminal tagged EscV with an 7L9A sequence instead of the original TMD6 in pSA10	This study
pEscV-TMD5_ex_-V5 (pSA10)	C-terminal tagged EscV with an 7L9A sequence instead of the original TMD5 in pSA10	This study
pEscV-TMD6_ex_-V5 (pSA10)	C-terminal tagged EscV with an 7L9A sequence instead of the original TMD6 in pSA10	This study
pEscV_G213A_-V5 (pSA10)	V5 C-terminal tagged EscV with a point mutation in position 213 in pSA10	This study
pEscV_G217A_-V5 (pSA10)	V5 C-terminal tagged EscV with a point mutation in position 217 in pSA10	This study
pEscV_G213L_-V5 (pSA10)	V5 C-terminal tagged EscV with a point mutation in position 213 in pSA10	This study
pEscV_G217L_-V5 (pSA10)	V5 C-terminal tagged EscV with a point mutation in position 217 in pSA10	This study
pToxR-GpA-MBP	The GpA TMD sequence inserted between ToxR and MBP	Langosch et al., 1996
pToxR-Tar1-MBP	Tar TMD1 sequence inserted between ToxR and MBP	Sal-Man et al., 2004
pToxR-A16-MBP	A sequence of 16 alanine residues inserted between ToxR and MBP	Langosch et al., 1996
pToxR-TMD1-MBP	TMD1 of EscV inserted between ToxR and MBP	This study
pToxR-TMD2-MBP	TMD2 of EscV inserted between ToxR and MBP	This study
pToxR-TMD3-MBP	TMD3 of EscV inserted between ToxR and MBP	This study
pToxR-TMD4-MBP	TMD4 of EscV inserted between ToxR and MBP	This study
pToxR-TMD5-MBP	TMD5 of EscV inserted between ToxR and MBP	This study
pToxR-TMD6-MBP	TMD6 of EscV inserted between ToxR and MBP	This study
pToxR-TMD7.1-MBP	TMD7.1 of EscV inserted between ToxR and MBP	This study
pToxR-TMD7.2-MBP	TMD7.2 of EscV inserted between ToxR and MBP	This study

### Bioinformatics

FASTA sequence for the EscV protein was downloaded from *GenBank* on the NCBI and UniProt database. To predict the TMD sequences, we used the TMHMM software^1^. ClustalW multisequence alignment algorithm was used to identify highly conserved protein domains by comparing sequences of EscV orthologs^2^ (no changes were made in the default parameters of the server; Larkin et al., 2007).

### Construction of Plasmids Expressing Labeled EscV

EscV-expressing plasmids were constructed using the primers presented in Table 2. The pSA10 plasmid was amplified using the primer pair pSA10_F/pSA10_R (Table 2). The *escV* gene was amplified from EPEC genomic DNA using the primer pairs EscV_SA10_F/EscV_His_R1 and then EscV_SA10_F/EscV_His_R2, which fused a His tag to the coding region of EscV (Table 2). The polymerase chain reaction (PCR) products were subjected to digestion with *Dpn*I, purified, and assembled by the Gibson assembly method (Gibson et al., 2008, 2009). An HA-labeled version of EscV was similarly cloned into pSA10; here, the double-HA tag was fused to the coding region of *escV* by amplification from EPEC genomic DNA using the primer pairs EscV_SA10_F/EscV_HA_R1 and then EscV_SA10_F/EscV_HA_R2 (Table 2). To clone EscV labeled with the V5 tag, we amplified the pEscD-V5 (pSA10) vector (Tseytin et al., 2018a) using the primer pair pSA10_V5_F/pSA10_R and the EPEC genomic DNA with EscV_SA10_F/EscV_V5_R (Table 2). The PCR products were subjected to digestion with *Dpn*I, purified, and assembled by the Gibson assembly. To clone EscV-V5 into a low-copy-number plasmid (pACYC184), we amplified the pEscP-V5 (pACYC184) vector (Shaulov et al., 2017) using the primer pair pSA10_V5_F/pACYC_R and the EPEC genomic DNA with EscV-V5_184_F/EscV-V5_184_R (Table 2). The PCR products were subjected to digestion with *Dpn*I, purified, and cloned using the Gibson assembly. The resulting constructs, pEscV_wt_-V5 (in pSA10 and pACYC184), pEscV_wt_-HA, and pEscV_wt_-His expressed a full-length EscV protein with V5, HA, or His tag fused to its C-terminal, respectively.

**TABLE 2:** Sequences of primers designed and used in this study.

Plasmid	Primer name	Primer sequence
pEscV_wt_-His (pSA10)	pSA10_F	AATTCCCGGGGATCCGTCG
	pSA10_R	CTGTTTCCTGTGTGAAATTGTTATCCG
	EscV_SA10_F	TCACACAGGAAACAGATGAATAAACTCTTAAATATATTTAAAAAAGCAG
	EscV_His_R1	TCAGTGGTGGTGTGCTCTGAAATCATTTACCGTTC
	EscV_His_R2	GATCCCCGGGAATTTCAGTGGTGGTGGTGGTGGTGTGCTCT
pEscV_wt_-2HA (pSA10)	EscV_HA_R1	GGGTAAGCGTAATCTGGAACATCGTATGGTGCTCTGAAATCATTTACCG
	EscV_HA_R2	GATCCCCGGGAATTTCAAGCGTAATCTGGAACATCGTATGGGTAAGCGTAATCTGG
pEscV-V5 (pSA10)	pSA10_V5_F	GGTAAGCCTATCCCTAACCCTC
	EscV_V5_R	GGTTAGGGATAGGCTTACCTGCTCTGAAATCATTTACCGTTC
pEscV-V5 (pACYC184)	pACYC_R	TTTTAAATTTTATTCATCCTGGTGGTTG
	EscV-V5_184_F	CCAGGATGAATAAAATTTAAAAATGAATAAACTCTTAAATATATTTAAAAAAGCAG
	EscV-V5_184_R	GGTTAGGGATAGGCTTACCTGCTCTGAAATCATTTACCGTTC
pEscV-TMD5_ex_-His (pSA10)	7L9A-F	CTGTTGCTACTCTTACTCCTTGCGGCCGCAGCGGCTGCAGCGGCAGCC
	7L9A-R	GGCTGCCGCTGCAGCCGCTGCGGCCGCAAGGAGTAAGAGTAGCAACAG
	EscV_TMD5-7L9A_F	GTATCATTATTGTTCTGTTGCTACTCTTACTCCTTG
	EscV_TMD5-7L9A_R	TCACTAAATGGCATGGCTGCCGCTGC
	EscV_Fc5_F	GGCAGCCATGCCATTTAGTGAGGCAC
	EscV_7L9A_Gib5_R	GTAGCAACAGAACAATAATGATACCAGCAATAGC
pEscV-TMD6_ex_-His (pSA10)	EscV_TMD5-7L9A_F	TTGGTTCTGTTGCTACTCTTACTCCTTGC
	EscV_TMD5-7L9A_R	GCACACGAGTGGCTGCCGCTGCAG
	EscV_Fc6_F	GCGGCAGCCACTCGTGTGCCCGG
	EscV_7L9A_Gib6_R	GAGTAGCAACAGAACCAAAGCATCACCGACAG
pEscV_G213A_-V5 (pSA10)	G213A_F	ACCTTTTTGGTGCCGTGCTCATTGGTATGTGG
	G213A_R	CCAATGAGCACGGCACCAAAAAGGTTAAC
pEscV_G217A_-V5 (pSA10)	G217A_F	CGTGCTCATTGCTATGTGGCAATTTGACATGC
	G217A_R	CAAATTGCCACATAGCAATGAGCACGCCAC
pEscV_G213L_-V5 (pSA10)	G213L_F	GTTAACCTTTTTGGTCTCGTGCTCATTGGTATGTGG
	G213L_R	CCAATGAGCACGAGACCAAAAAGGTTAACTAAAAC
pEscV_G217L_-V5 (pSA10)	G217L_F	GGCGTGCTCATTCTTATGTGGCAATTTGACATGC
	G217L_R	CAAATTGCCACATAAGAATGAGCACGCCACCAAAAAGG
pToxR_TMD1_MBP	EscV_TMD1_F	CTAGCATTCTGGCTCTCTTCTTCTTTATGGCTGTAATGATGATGATTATTCCAGG
	EscV_TMD1_R	GATCCCTGGAATAATCATCATCATTACAGCCATAAAGAAGAAGAGAGCCAGAATG
pToxR_TMD2_MBP	EscV_TMD2_F	CTAGCGCGATTAATATTTCGACAGCTTTACTTTTATTAATGCTCTCAATCTATATAGG
	EscV_TMD2_R	GATCCCTATATAGATTGAGAGCATTAATAAAAGTAAAGCTGTCGAAATATTAATCGCG
pToxR_TMD3_MBP	EscV_TMD3_F	CTAGCATCCTGTTGATTACGACGTTGATGCGCCTGTCGCTCAGTGTTAGTACAGG
	EscV_TMD3_R	GATCCCTGTACTAACACTGAGCGACAGGCGCATCAACGTCGTAATCAACAGGATG
pToxR_TMD4_MBP	EscV_TMD4_F	CTAGCGGCCTGGTTATTTTTACTATTATCACCATTGTTCAATTTATGGTTATTGG
	EscV_TMD4_R	GATCCCAATAACCATAAATTGAACAATGGTGATAATAGTAAAAATAACCAGGCCG
pToxR_TMD5_MBP	EscV_TMD5_F	CTAGCTTAGTTAACCTTTTTGGTGGCGTGCTCATTGGTATGTGGCAATTTGACGG
	EscV_TMD5_R	GATCCCGTCAAATTGCCACATACCAATGAGCACGCCACCAAAAAGGTTAACTAAG
pToxR_TMD6_MBP	EscV_TMD6_F	CTAGCGCCCAGATCCCTGCGCTTATTATTTCTGTCACCGCGGGCGTGGTTGTTGG
	EscV_TMD6_R	GATCCCAACAACCACGCCCGCGGTGACAGAAATAATAAGCGCAGGGATCTGGGCG
pToxR_TMD7.1_MBP	EscV_TMD7.1_F	CTAGCGCGATTATTCCGGGCTTTCCTACATTGGTCTTCTTATTTCTGGCGGTTGG
	EscV_TMD7.1_R	GATCCCAACCGCCAGAAATAAGAAGACCAATGTAGGAAAGCCCGGAATAATCGCG
pToxR_TMD7.2_MBP	EscV_TMD7.2_F	CTAGCCCTACATTGGTCTTCTTATTTCTGGCGGTTTGCCTGTTGGGGATAGCCGG
	EscV_TMD7.2_R	GATCCCGGCTATCCCCAACAGGCAAACCGCCAGAAATAAGAAGACCAATGTAGGG

The TMD5-exchanged and TMD6-exchanged *escV* in pSA10 were generated by using the template of pEscV_wt_-His (pSA10). To replace the TMD5 of EscV by a TMD backbone sequence of 7-leucine-9-alanine (7L9A), the EscV 222–675 amino acid sequence was amplified by using the primer pair EscV_Fc5_F/EscV_His_R2 (Table 2) from the pEscV_wt_-His vector. The TMD 7L9A backbone was generated by annealing the primer pair 7L9A-F/7L9A-R (Table 2) by heating the sample to 95°C for 5 min and then decreasing the temperature to 20°C at a rate of 5°C/min. Then the 7L9A backbone was amplified by the primer pair EscV_TMD5-7L9A_F/EscV_TMD5-7L9A_R (Table 2). The EscV_222__–__675_ PCR fragment and the 7L9A backbone were then ligated by using overlapping sequences and amplified by using the primer pair EscV_TMD5-7L9A_F/EscV_His_R2 (Table 2). The Gibson assembly was conducted by amplifying the pEscV_wt_-His pSA10 vector with the primer pair pSA10_F/EscV_7L9A_Gib5_R (Table 2), followed by treating the reaction with *Dpn*I and subjecting the amplified vector and the 7L9A-EscV_222__–__675_-fused PCR fragment to ligation. The resulting construct, pEscV-TMD5_ex_-His (pSA10), expressed a TMD5-exchanged EscV with a His tag at its C-terminus. The TMD6-exchanged *escV* in pSA10 was generated by a similar manner. The TMD 7L9A backbone was amplified by the primer pair EscV_TMD6-7L9A_F/EscV_TMD6-7L9A_R and fused to the EscV_259__–__675_ PCR fragment, which was amplified by using the primer pair EscV_Fc6_F/EscV_His_R2 (Table 2). The Gibson assembly was conducted by amplifying the pEscV_wt_-His pSA10 vector with the primer pair pSA10_F/EscV_7L9A_Gib6_R (Table 2), followed by treating the reaction with *Dpn*I and subjecting the amplified vector and the 7L9A-EscV_259__–__675_-fused PCR fragment to ligation. The resulting construct, pEscV-TMD6_ex_-His (pSA10), expressed a TMD6-exchanged EscV with a His tag at its C-terminus. To move the TMD5-exchanged and TMD6-exchanged EscV into pSA10 that fuses the proteins with the V5 tag, pEscV-TMD5_ex_-His and pEscV-TMD6_ex_-His were amplified using the primer pair EscV_SA10_F/EscV_V5_R, and the pEscV_wt_-V5 vector was amplified by the primer pair pSA10_V5_F/pSA10_R (Table 2). The PCR products were treated with *Dpn*I and subjected to Gibson assembly.

Site-directed mutagenesis of G213A, G217A, G213L, and G217L within the EscV-V5 (pSA10) construct was performed using the primer pairs G213A_F/G213A_R, G217A_F/G217A_R, G213L_F/G213L_R, and G217L_F/G217A_L (Table 2). All constructs were verified by DNA sequencing.

### Detection of the Homo-Oligomerization of TMDs Within the Membrane

The ToxR transcription activator can be successfully used to detect TMD–TMD interactions (Brosig and Langosch, 1998). DNA cassettes, encoding single TMDs of EscV (TMD1–TMD7.2), glycophorin A (GpA) TMD, *E. coli* aspartate receptor N-terminal TMD (Tar-1), 16-alanine backbone (A16), and no TMD (ΔTMD), were grafted between the cytoplasmic domain of the ToxR transcription activator protein (an oligomerization-dependent transcriptional activator) and the periplasmic domain of the maltose-binding protein (MBP). This was performed by aligning the oligonucleotides pairs encoding a *Nhe*I–*Bam*HI TMD-DNA cassette of 16 core residues of the EscV TMD1–TMD7 (Table 2). The double-stranded fragments were oligophosphorylated and ligated between the *toxR* transcription activator and the *malE* (encodes *E. coli* MBP) within a *Nhe*I–*Bam*HI digested ToxR-MBP plasmid (Table 2).

The MBP domain directs the chimera protein to the periplasm (Salema and Fernandez, 2013) where the TMD becomes embedded within the inner membrane. In the assay, we transform ToxR-TMD-MBP plasmids containing different TMDs into *E. coli* FHK12 cells, which contain a reporter gene, coding for β-galactosidase, under the control of the *ctx* promoter (Kolmar et al., 1995). Oligomerization of the investigated TMD derives ToxR oligomerization, which then can bind (in its oligomeric form) the *ctx* promoter and transcribe the reporter gene *lacZ* (Ottemann et al., 1992; Brosig and Langosch, 1998). Determination of the oligomerization level is calculated from the activity of β-galactosidase that is measured by the levels of a yellow color (OD_405_) produced from the cleavage of the *o-*nitrophenylgalactose (ONPG), β-galactosidase substrate (Langosch et al., 1996; Fink et al., 2012). We monitored the activity of β-galactosidase for 20 min, at intervals of 30 s and calculated the *V*_max_ of the reaction. These were normalized to the original cell content (measured at OD_600_) and are presented in Miller units. We used the GpA TMD sequence as a positive control for strong homo-oligomerization (Russ and Engelman, 2000), the N-terminal TMD of the *E. coli* aspartate receptor (Tar-1) as a reference for moderate oligomerization (Sal-Man et al., 2004), and the A16 sequence as a control for non-oligomerizing sequences (Bronnimann et al., 2013).

### Maltose Complementation Assay

Membrane localization and correct orientation of the chimera proteins were examined as described previously (Langosch et al., 1996). Briefly, we transformed PD28 cells, *E. coli* strain deficient for *malE* (Duplay and Szmelcman, 1987), with the different ToxR-TMD-MBP constructs described above. The bacteria were grown overnight, washed, and then inoculated into M9 minimal medium supplemented with 0.4% maltose. Bacterial growth (OD_600_) was measured over time. Since PD28 cells are unable to utilize maltose, only strains that translocate the chimera protein across the inner membrane were expected to grow in this medium. A construct without TMD (ΔTMD) served as a negative control. This chimera protein is expected to reside in the bacterial cytoplasm and, therefore, cannot grow in minimal medium.

### *In vitro* Type 3 Secretion Assay

Type 3 secretion assays were performed as previously described (Tseytin et al.,2018a,b, 2019). Briefly, overnight EPEC strains grown in LB were diluted 1:40 into preheated DMEM and grown for 6 h at 37°C in an atmosphere of 5% CO_2_, statically, to an optical density of 0.7 (OD_600_). Protein expression was induced by 0.1 mM IPTG. To separate bacterial cells from bacterial supernatants, we centrifuged the cultures at 20,000 × *g* for 5 min; the bacterial pellets were dissolved in an sodium dodecyl sulfate (SDS)-polyacrylamide gel electrophoresis (PAGE) sample buffer, and the supernatants, containing secreted proteins, were collected and filtered through a 0.22-μm filter (Millipore). The supernatants were normalized according to the bacterial OD_600_ and precipitated with 10% (v/v) trichloroacetic acid (TCA) overnight at 4°C. To concentrate the secreted proteins, the supernatant samples were centrifuged at 20,000 × *g* for 30 min at 4°C and resuspended in the SDS-PAGE sample buffer. To neutralize the residual TCA, we added saturated Tris.

### Immunoblotting

Samples were subjected to 12% SDS-PAGE and transferred to nitrocellulose (pore size, 0.45 μm; Bio-Rad) or polyvinylidene difluoride (PVDF; Mercury; Millipore) membranes. The blots were blocked with 5% (w/vol) skim milk–PBST (0.1% Tween in phosphate-buffered saline) for 1 h; then incubated with the primary antibody for 1 h at room temperature, or overnight at 4°C; washed with PBST three times; and then incubated with the secondary antibody (diluted in 5% skim milk–PBST, for 1 h, at room temperature). Chemiluminescence was detected with the EZ-ECL reagents (Biological Industries). A dilution of 1:1,000 was used for mouse anti-HA (Abcam), anti-V5 (Invitrogen), anti-His (Thermo Fisher Scientific), anti-HSV (Novagen), anti-actin (MPBio), anti-JNK (BD Pharmingen), anti-DnaK (Abcam), and rabbit anti-MBP (Thermo Fisher Scientific). Mouse anti-intimin (a gift from B. Brett Finlay, UBC) was diluted 1:2,000 (Gauthier et al., 2003), and horseradish peroxidase (HRP)-conjugated goat anti-mouse antibody (Abcam) and HRP-conjugated goat anti-rabbit antibody (Abcam) were diluted 1:10,000.

### Translocation Activity

Translocation assays were performed as previously described (Baruch et al., 2011). Briefly, EPEC strains were pre-induced for 3 h for T3SS activity (preheated DMEM, statically, in a CO_2_ tissue culture incubator) and then were used to infect HeLa cells (8 × 10^5^cells per well) at a multiplicity of infection (MOI) of 1:300 for 3 h. Cells were then washed and lysed with RIPA buffer [10 mM Tris-Cl pH 8.0, 1 mM EDTA, 0.5 mM EGTA, 1% Triton X-100, 0.1% sodium deoxycholate, 0.1% SDS, 140 mM NaCl; before use, add 1 mM phenylmethylsulfonyl fluoride (PMSF)]. Lysates were centrifuged at maximum speed for 5 min to remove non-lysed cells and supernatants containing cellular proteins, were collected, mixed with the SDS-PAGE sample buffer, and analyzed by western blot analysis with anti-JNK and anti-actin antibodies (loading control). Uninfected HeLa cell samples, and HeLa cells infected by EPEC Δ*escN* and EPEC Δ*escV* mutant strains were used as negative controls.

### Bacterial Fractionation

Bacterial cell fractionation was performed as previously described (Gauthier et al., 2003). Briefly, overnight EPEC cultures were subcultured at 1:50 in DMEM and grown for 6 h, at 37°C, in a CO_2_ tissue culture incubator. Bacteria were collected, washed, and resuspended in buffer A [50 mM Tris (pH 7.5), 20% (w/v) sucrose, 5 mM EDTA, protease inhibitor cocktail (Roche Applied Science), and lysozyme (100 μg/mL)] to generate spheroplasts. The samples were incubated for 15 min, at room temperature, and while rotating, MgCl_2_ (20 mM) was then added. The samples were spun for 10 min at 5,000 × *g*, and the supernatants, containing the periplasmic fractions, were collected. The pellets were resuspended in lysis buffer (20 mM Tris/HCl pH 7.5, 150 mM NaCl, 3 mM MgCl_2_, 1 mM CaCl_2_, and 2 mM β-mercaptoethanol with protease inhibitors) and kept at 4°C from this step on. The samples were sonicated (Thermo Fisher Scientific, 3 × 10 s) after addition of RNase A and DNase I (10 μg/ml) and centrifuged (2,300 × *g* for 15 min) to remove intact bacteria, and the supernatants, containing cytoplasmic and membrane proteins, were collected. To separate the cytoplasmic and membrane fractions, the samples were centrifuged (in a Beckman Optima XE-90 Ultracentrifuge with a SW60 Ti rotor) for 30 min at 100,000 × *g*. The supernatants, containing the cytoplasmic fraction, were collected, and the pellets, containing the membrane fractions, were washed with lysis buffer, and the final pellets were resuspended in lysis buffer with 0.1% SDS. We evaluated the protein content by Coomassie Plus protein assay. We used various proteins as membrane (Intimin), periplasm (MBP), and cytoplasm (DnaK) markers.

### Crude Membrane

We isolated the bacterial membrane fraction using the bacterial fractionation protocol described above. To extract membrane proteins from the crude membranes, samples were resuspended in lysis buffer containing 1% *n*-dodecyl-β-D-maltoside (DDM) and incubated for 60 min on a rotary wheel, at 4°C. The samples were then centrifuged (20,000 × *g*, for 15 min, at 4°C) to remove non-solubilized material, and the supernatants, containing membrane proteins, were collected and analyzed by blue-native (BN)-PAGE.

### Blue-Native-PAGE

We incubated the extracted membrane proteins for 15 min in a BN sample buffer (30% glycerol with 0.05% Coomassie Brilliant Blue G250), and then loaded the samples onto a Criterion XT Tris-Acetate 3–8% precast gradient native gel (Bio-Rad). Electrophoresis was carried out on ice using a cathode buffer (15 mM Bis-Tris and 50 mM bicine, pH 7) and anode buffer (50 mM Bis-Tris, pH 7) until full separation (5–6 h). The gel was then incubated in a transfer buffer and subjected to western immunoblotting.

## Results

**FIGURE 1:**
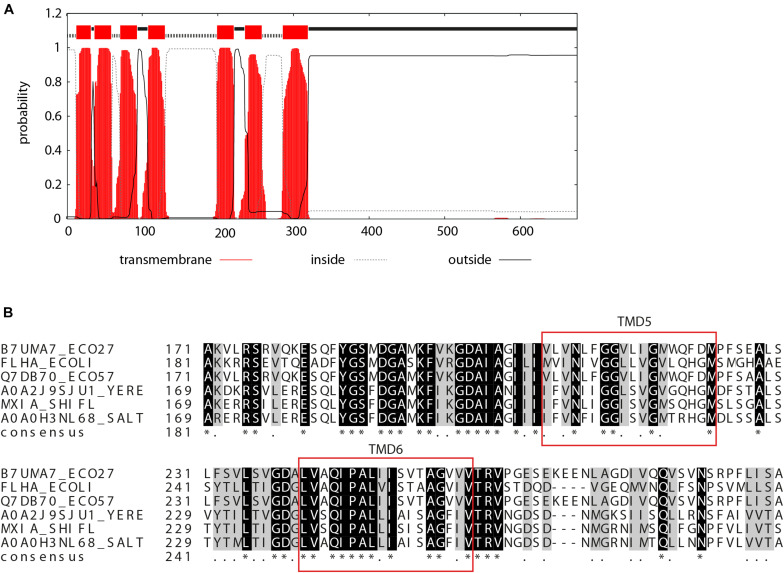
Prediction of TMDs of EscV. **(A)** TMHMM software prediction analysis of the probability of each EscV amino acid to be localized within the bacterial membrane. Seven TMDs were identified (sequence is colored red). **(B)** Sequence alignment of the EscV export apparatus protein. A standard protein BLAST alignment is presented by ClustalW (Larkin et al., 2007) for EscV of EPEC T3SS (B7UMA7), FlhA of *E. coli* flagella (P76298), EscV of the *E. coli* O157:H7 (Q7DB70), YscV of the *Y. enterocolitica* (A0A2J9SJU1), MxiA of the *Shigella* T3SS (P0A1I5), and InvA of the *S. typhimurium* T3SS (A0A0H3NL68). A high level of conservation was observed within the TMD6 sequence and for the GxxxG motif found within TMD5.

The soluble C-terminal region of EscV and its homologs is well characterized (Abrusci et al., 2013; Majewski et al., 2020), yet not much is known about the N-terminal region, which is predicted to be embedded within the bacterial membrane. To identify EscV TMDs, we analyzed EscV’s sequence using TMD prediction software (TMPred, TMHMM, and Phobius) and found seven regions with high probability to serve as TMDs: TMD1, residues 17–39; TMD2, residues 43–62; TMD3, residues 74–96; TMD4, residues 111–133; TMD5, residues 205–227; TMD6, residues 237–259; and TMD7, residues 296–329 (Figure 1A). To identify conserved motifs/residues within EscV TMDs, we performed multiple sequence alignment of EPEC EscV (B7UMA7), FlhA of *E. coli* flagella (P76298), EscV of the *E. coli* O157:H7 (Q7DB70), YscV of the *Yersinia enterocolitica* (A0A2J9SJU1), MxiA of the *Shigella* T3SS (P0A1I5), and InvA of the *S. typhimurium* T3SS (A0A0H3NL68) by Clustal Omega and presented them using the BoxShade software (Supplementary Figure 1). Among the TMDs, we found that TMD6 showed the highest sequence conservation, with 65% identity (Figure 1B). In addition, we found that TMD5 contains a GxxxG motif, which was previously reported to be critical for TMD–TMD interactions within the membrane (Moore et al., 2008).

### EscV TMD5 and TMD6 Support TMD–TMD Interactions

**FIGURE 2:**
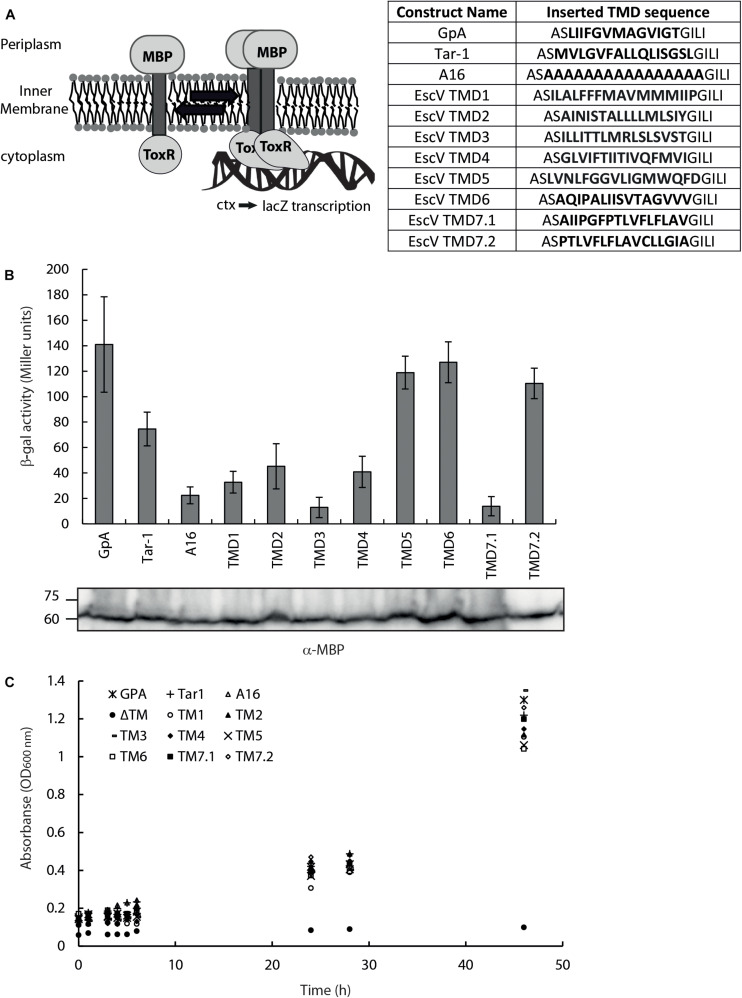
EscV TMD self-oligomerization activity. **(A)** Schematic illustration of a ToxR assembly system. TMD–TMD interaction promotes oligomerization of the transcription activator ToxR, which then can bind (in its oligomeric form) the *ctx* promoter and transcribe the reporter gene, *lacZ*. The TMD sequences inserted between the ToxR and the MBP are presented. **(B)** The LacZ activities of FHK12 bacterial strains expressing the ToxR-TMD-MBP chimeras of various EscV TMDs, GpA, Tar-1, and A16 TMDs. Bars represent the standard deviation of at least three independent experiments. The expression of ToxR-TMD-MBP chimera proteins containing the different TMD sequences was analyzed on SDS-PAGE and western blotting using an anti-MBP antibody and presented under each corresponding sample. **(C)** Growth curves of PD28 bacteria transformed with plasmids encoding ToxR-TMD-MBP chimera protein containing the GpA (*), Tar-1 (+), A16 (△), EscV TMD1 (∘), TMD2 (▲), TMD3 (-), TMD4 (◆), TMD5 (×), TMD6 (□), TMD7.1 (■), TMD7.2 (◆) or in the absence of a TMD (ΔTM,^•^). The bacteria were grown in a minimal medium containing maltose. All bacterial cultures showed similar growth curves, indicating proper membrane integration.

As TMDs are known to be involved in protein–protein interactions, we examined the ability of isolated EscV TMDs to support self-interaction. For that purpose, we employed the ToxR assembly system (Figure 2A), which monitors the strength of TMD–TMD interactions within the bacterial inner membrane (Langosch et al., 1996; Joce et al., 2011). We compared the oligomerization level of EscV TMDs with that of GpA’s TMD sequence, which contains a GxxxG motif and is used as a reference for strong homo-oligomerization (Lemmon et al., 1992; Adair and Engelman, 1994; Russ and Engelman, 2000). We also compared the EscV TMD oligomerization levels with the N-terminal TMD of the *E. coli* aspartate receptor (Tar-1), which has moderate oligomerization (Sal-Man et al., 2004), and polyalanine’s (A16) sequence as a non-oligomerizing sequence (Langosch et al., 1996; Sal-Man et al., 2005). Since the amino acid sequence of TMD7 was significantly longer than that of the other TMDs and the ToxR system responded differently to various TMD lengths (Langosch et al., 1996), we decided to test two different forms of this TMD, TMD7.1, and TMD7.2, which are of a similar length as the other examined TMDs. The sequences of the TMDs studied are presented in Figure 2A. We observed strong TMD self-oligomerization activity for EscV’s TMD5, TMD6, and TMD7.2 compared to the activities of the GpA and Tar-1 TMDs, whereas EscV’s TMD1, TMD2, TMD3, TMD4, and TMD7.1 showed reduced oligomerization activities compared to GpA (Figure 2B). As expected, the oligomerization of the A16 background control was low (Figure 2B). These findings suggested that TMD5, TMD6, and TMD7.2 of EscV might be involved in the oligomerization of the full-length protein EscV, through TMD–TMD interactions. To exclude the possibility that the high self-oligomerization activity of EscV’s TMD5, TMD6, and TMD7.2 resulted from higher expression levels of these chimera proteins, we subjected the bacterial samples to SDS-PAGE and western immunoblotting analysis with an anti-MBP antibody. All samples showed comparable expression levels (Figure 2B). To verify that the ToxR-TMD-MBP chimera proteins correctly integrated into the inner membrane, we employed the maltose complementation assay. For that purpose, we used an *E. coli* strain with a *malE* gene (PD28) deletion, which cannot produce endogenous MBP and therefore cannot support bacterial growth in minimal medium with maltose as the sole carbon source (Langosch et al., 1996). Only strains that express the chimera protein ToxR-TMD-MBP and orient it across the inner membrane, with MBP facing the periplasm, will support bacterial growth. We observed that all examined strains demonstrated bacterial growth, which indicated proper membrane integration, while the negative control that did not contain a TMD (ΔTMD) showed no growth, as expected (Figure 2C). Overall, these results suggest that TMD5, TMD6, and TMD7.2 of EscV are involved in EscV self-oligomerization through TMD–TMD interactions. However, due to the high conservation of TMD6 and the GxxxG motif within TMD5, on one hand, and the unclear boundaries of TMD7, on the other, we decided to focus on EscV’s TMD5 and TMD6.

### Replacement of EscV TMDs With a Non-oligomerizing Sequence (7L9A) Affects Bacterial Fitness

**FIGURE 3:**
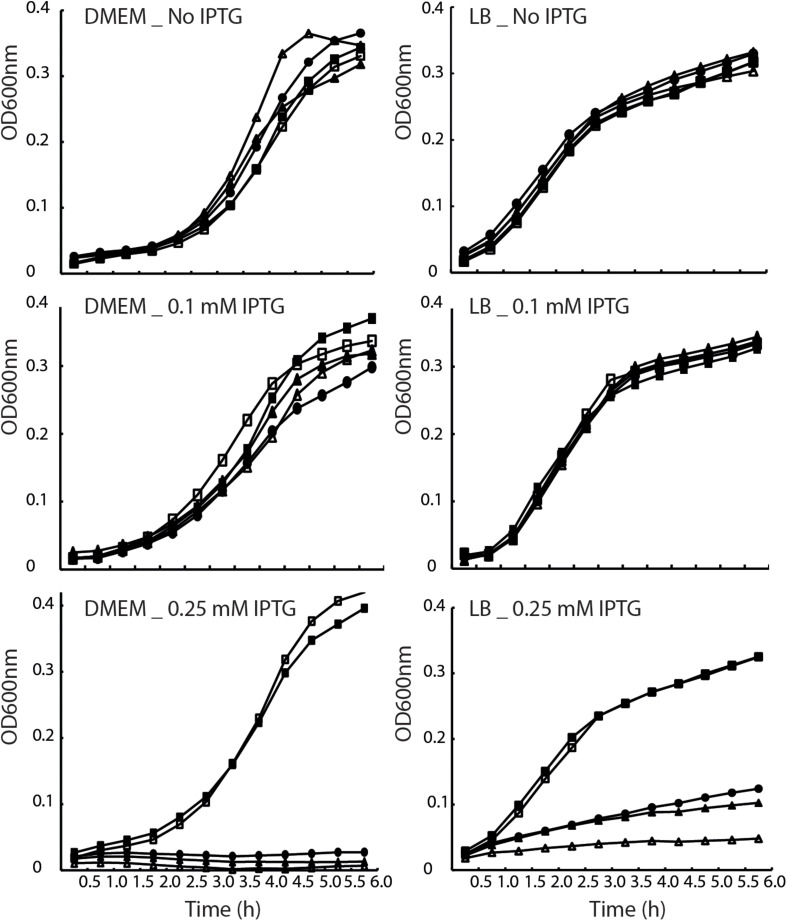
Overexpression of EscV reduces bacterial fitness. Growth curves of WT EPEC (■), Δ*escV* (□), and EPEC Δ*escV* transformed with EscV_wt_-His (^•^), EscV-TMD5_ex_-His (△), EscV-TMD6_ex_-His (▲). Strains were grown at 37°C in DMEM (left panel) and LB (right panel) media with various IPTG concentrations (0, 0.1, and 0.25 mM). Optical density at 600 nm was measured every 30 min and plotted over time.

To examine whether EscV’s TMD5 and TMD6 serve solely as membrane anchors or have a functional role within the full-length protein, we constructed EscV mutant proteins lacking TMD5 or TMD6 sequences. Since EscV lacking its TMD5 or TMD6 will likely adopt alternate protein folding compared to the native protein or have impaired localization, we constructed TMD5- and TMD6-exchanged EscV proteins, where the native core TMD5 and TMD6 sequences (16 amino acids in length) were replaced by a hydrophobic sequence. We chose a hydrophobic sequence of seven consecutive leucine residues followed by nine alanine residues (7L9A), which was previously shown to be sufficiently hydrophobic to support protein integration into the membrane yet cannot support TMD–TMD interactions (Sal-Man et al., 2005). To determine the biological effect of this replacement, we transformed the TMD5- and TMD6-exchanged EscV (EscV-TMD5_ex_-His and EscV-TMD6_ex_-His), as well as EscV_wt_-His, into the *escV*-null strain (Δ*escV*) and examined their ability to restore T3SS activity. However, when EscV overexpression was induced by addition of IPTG to a concentration of 0.25 mM, growth rate was reduced in all strains (Figure 3). To determine the conditions that allow EscV expression without severe reduction of bacterial fitness, we grew WT EPEC, EPEC Δ*escV*, and EPEC Δ*escV* carrying either pEscV_wt_-His, pEscV-TMD5_ex_-His, or pEscV-TMD6_ex_-His in LB or in DMEM (which is used for T3SS-inducing conditions), in the presence (0.1 or 0.25 mM) or the absence of IPTG. Optical density at 600 nm was measured over time (Figure 3). We observed that expressions of EscV WT and TMD-exchanged versions have reduced fitness when induced with IPTG at a concentration higher than 0.1 mM (Figure 3). These results suggest that overexpression of EscV is toxic to bacteria and therefore negatively affects bacterial growth. Based on these results, we used 0.1 mM IPTG for our experiments.

### TMD5 and TMD6 Are Critical for EscV Activity

To examine whether EscV TMD5 and TMD6 sequences are critical for the activity of the full-length protein, we examined whether EscV-TMD5_ex_-His and EscV-TMD6_ex_-His can restore the T3SS activity of the EPEC Δ*escV* strain. Only functional EscV can restore the T3SS of the Δ*escV* strain, which is measured by the ability of EPEC strains to secrete three T3SS translocators (EspA, EspB, and EspD) into the culture supernatant, when grown under T3SS-inducing conditions.

**FIGURE 4:**
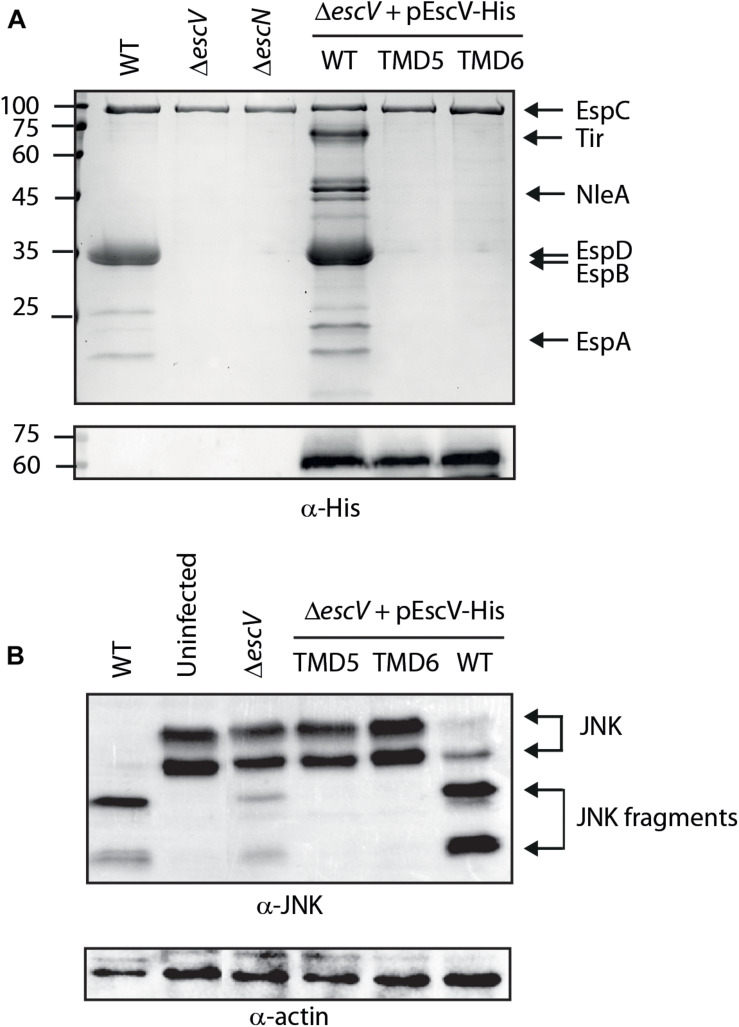
Replacement of EscV TMD5 and TMD6 by an alternative hydrophobic sequence abolishes T3SS activity. **(A)** Protein secretion profiles of EPEC WT, Δ*escV*, Δ*escN* and EPEC Δ*escV* strain carrying the pEscV_wt_-His, pEscV-TMD5_ex_-His, or pEscV-TMD6_ex_-His plasmids grown under T3SS-inducing conditions with 0.1 mM IPTG. The secreted fractions were filtered and protein content was concentrated from the supernatants of bacterial cultures and analyzed by SDS-PAGE and Coomassie blue staining. The T3SS-secreted translocators and effectors EspA, EspB, EspD, NleA, and Tir are marked on the right of the gel. EspC, which is not secreted via the T3SS, is also indicated. EscV expression was examined by analyzing bacterial pellets by SDS-PAGE and western blot analysis with an anti-His antibody. Numbers on the left are molecular masses in kilodaltons. **(B)** Proteins extracted from HeLa cells infected with WT, Δ*escN*, Δ*escV*, or Δ*escV* carrying the pEscV_wt_-His, pEscV-TMD5_ex_-His, or pEscS-TMD6_ex_-His, were subjected to western blot analysis using anti-JNK antibody and anti-actin (loading control). JNK and its degradation fragments are indicated.

First, we evaluated the ability of WT EscV to restore the T3SS activity of Δ*escV*. We observed that the expression of EscV_wt_-His within the Δ*escV* strain restored secretion of translocators but also resulted in hypersecretion of effectors (Tir and NleA; Figure 4A). To evaluate whether this phenotype occurs due to the labeling of EscV or to its expression from a plasmid, we examined the T3SS activity of Δ*escV*-carrying plasmids with unlabeled EscV or EscV labeled with various tags and expressed from low- and high-copy-number plasmids. We observed that transformation of unlabeled EscV_wt_ resulted in a milder phenotype and that only a slight elevation in effector secretion was observed. In contrast, expression of labeled EscV, regardless of the tag type, resulted in hypersecretion of effectors (Supplementary Figure 2). Interestingly, expressions of both EscV-TMD5_ex_-His and EscV-TMD6_ex_-His failed to complement the T3SS activity of the Δ*escV* strain and demonstrated a secretion profile similar to that of Δ*escV* and Δ*escN* (Figure 4A). Comparable protein expression of the WT and the exchanged versions was observed by analyzing whole-cell lysates by western blot analysis using anti-His antibody (Figure 4A).

To analyze whether the unregulated secretion of Δ*escV* transformed with pEscV_wt_-His affected the ability of the bacteria to infect host cells, we examined the ability of the strain to infect and translocate effectors into the HeLa cells. For this purpose, we infected HeLa cells with WT, Δ*escN*, Δ*escV*, and Δ*escV* transformed with pEscV_wt_-His and examined the cleavage pattern of JNK, a cellular protein that is cleaved by NleD, a translocated EPEC effector (Baruch et al., 2011). WT EPEC induced extensive degradation of JNK, as expected, relative to the uninfected sample and to the samples infected with Δ*escN* or Δ*escV* mutant strains (Figure 4B). EPEC Δ*escV* transformed with the plasmid encoding EscV_wt_-His showed a JNK degradation profile, indicating functional complementation by His-labeled EscV (Figure 4B). In addition, the Δ*escV* strain transformed with EscV TMD-exchanged versions (pEscV-TMD5_ex_-His or pEscV-TMD6_ex_-His) showed no degradation of JNK, as observed for the uninfected sample (Figure 4B). Overall, our results suggest that His-labeled EscV functionally complements the T3SS activity; however, replacing the native TMD5 or TMD6 sequences of EscV with an alternative hydrophobic sequence (7L9A) impairs the function of the T3SS (Figure 4B).

### Transmembrane Domain Replacement Does Not Affect EscV Localization to the Bacterial Membrane

**FIGURE 5:**
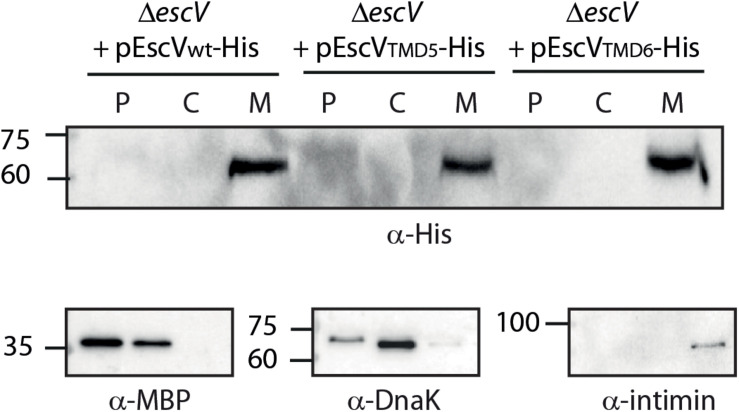
Replacement of EscV TMDs by an alternative hydrophobic sequence does not affect membrane localization. EPEC Δ*escV* strain expressing EscV_wt_-His, EscV-TMD5_ex_-His and EscV-TMD6_ex_-His were grown under T3S-inducing conditions, were fractionated into periplasmic (P), cytoplasmic (C), and membrane (M) fractions and analyzed by western blot analysis with an anti-His antibody. Proper bacterial fractionation was confirmed by analyzing the samples by SDS-PAGE and western blotting with anti-DnaK (cytoplasmic marker), anti-MBP (periplasmic marker), and anti-intimin (membrane marker) antibodies.

To exclude the possibility that EscV-TMD5_ex_-His and EscV-TMD6_ex_-His failed to complement the Δ*escV* T3SS activity due to impaired subcellular localization, we grew the strains under T3SS-inducing conditions and fractionated them into periplasmic, cytoplasmic, and membrane fractions. Our results showed that EscV-TMD5_ex_-His and EscV-TMD6_ex_-His localized mostly to the membrane fraction, as was seen for EscV_wt_-His (Figure 5). Correct bacterial fractionation was confirmed by analyzing the samples with anti-MBP (periplasmic marker), anti-DnaK (cytoplasmic marker), and anti-intimin (membrane marker) antibodies. Overall, our results indicated that replacement of TMD5 and TMD6 did not disrupt EscV localization to the bacterial membrane.

### EscV TMD6 Is Involved in Complex Formation

**FIGURE 6:**
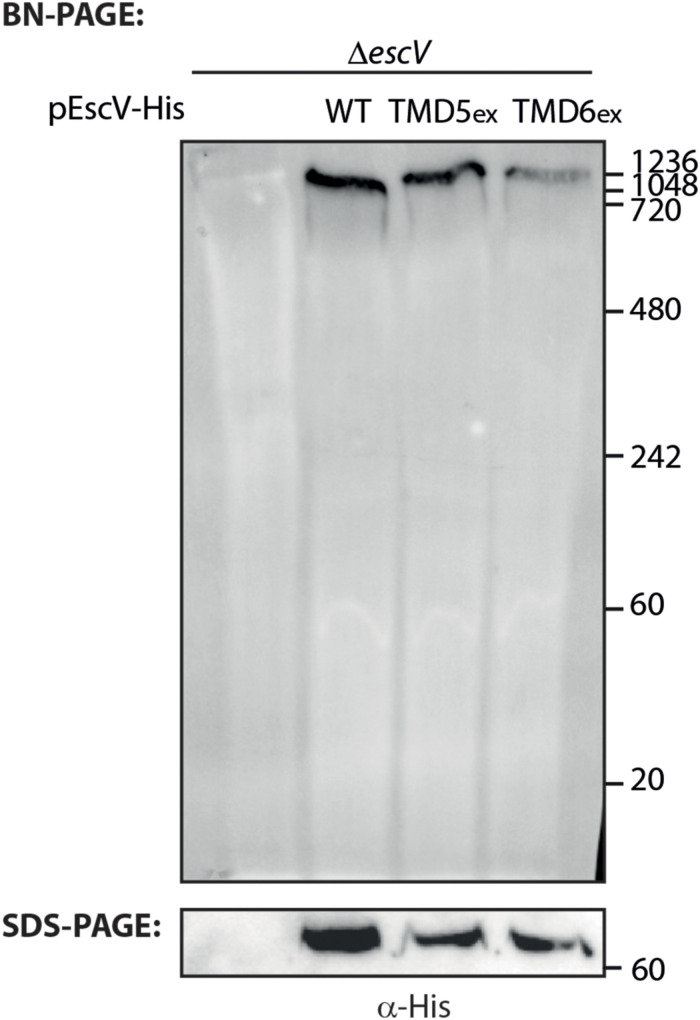
Association of EscV-exchanged version with the T3SS complex. Membrane protein extracts of Δ*escV*, Δ*escV* expressing EscV-His, EscV-TMD5_ex_-His, and EscV-TMD6_ex_-His were incubated in BN sample buffer, subjected to BN-PAGE (upper panel) and SDS-PAGE (lower panel), and western blot analysis using anti-His antibodies.

To investigate whether the EscV TMD-exchanged variants fail to complement the T3SS activity of the Δ*escV*-null strain due to their inability to properly integrate into the T3SS complex, we prepared crude membrane samples of EPEC Δ*escV* and EPEC Δ*escV*-null strains transformed with EscV_wt_-His, EscV-TMD5_ex_-His, and EscV-TMD6_ex_-His grown under T3SS-inducing conditions. The samples were then analyzed by BN-PAGE and immunoblotting. BN-PAGE analysis revealed that EscV_wt_-His and EscV-TMD5_ex_-His preserved the ability to integrate into the T3SS complex, as they migrated primarily as a large complex (>1 MDa) to the top of the gel. However, EscV-TMD6_ex_-His integration into the complex appeared to be impaired (Figure 6). To verify that the modified running pattern of the EscV TMD6-exchanged version was not due to reduced protein expression, we analyzed the crude membrane extracts by SDS-PAGE and western blotting using anti-His antibody. Similar expression levels were observed for all EscV variants (Figure 6). These results suggest that TMD5 and TMD6 are not critical for the integration of EscV into the T3SS complex, as EscV-exchanged versions enabled the formation of high-molecular-weight complexes. EscV-TMD5_ex_-His fully preserved the ability to integrate into the full or intermediate T3SS complexes, while integration of EscV-TMD6_ex_-His was impaired.

### A Single Mutation Within the GxxxG Motif of TMD5 Abolished EPEC T3SS Activity and Complex Formation

**FIGURE 7:**
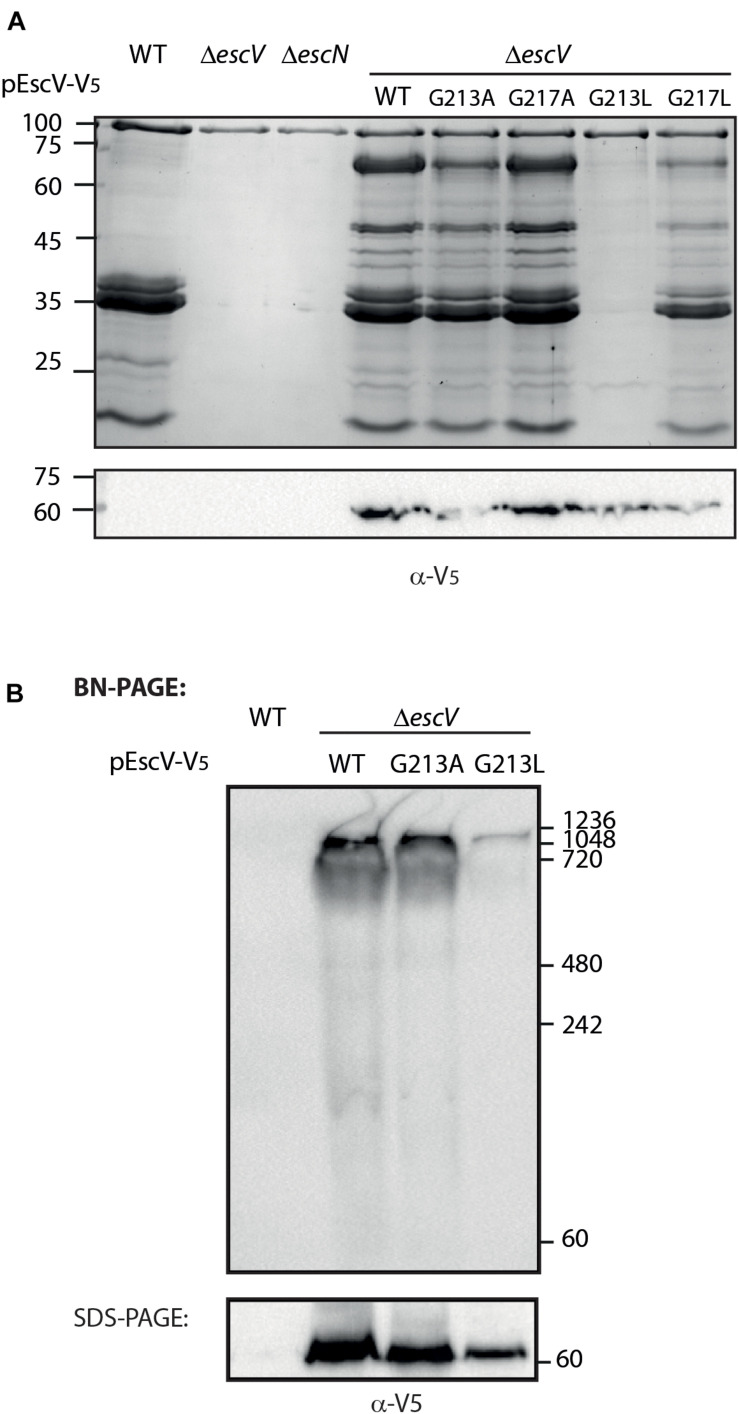
The glycine residue at position 213 is critical for the T3SS activity. **(A)** Protein secretion profiles of EPEC strains grown under T3SS-inducing conditions: WT, Δ*escN*, Δ*escV*, and Δ*escV* complemented with EscV_wt_-V5, EscV_G213A_-V5, EscV_G217A_-V5, EscV_G213L_-V5, or EscV_G217L_-V5. The secreted fractions were concentrated from the supernatants of bacterial cultures and analyzed by SDS-PAGE and Coomassie blue staining. The expression of EscV-V5 variants were examined by analyzing the bacterial pellets by SDS-PAGE and western blot analysis with an anti-V5 antibody. **(B)** Membrane protein extracts of WT EPEC and Δ*escV* expressing EscV_wt_-V5, EscV_G213A_-V5, or pEscV_G213L_-V5 were incubated in BN sample buffer and then subjected to BN-PAGE (upper panel) or SDS-PAGE (lower panel) and western blot analysis using anti-V5 antibody. Molecular masses in kilodaltons are presented on the right.

To examine whether the GxxxG motif identified within TMD5 is critical for protein activity, we mutated the glycine residues at positions 213 and 217 to either alanine or leucine (G213A, G217A, G213L, and G217L). Due to expression challenges of the mutated proteins tagged with His-tag, we labeled EscV WT and single mutants with the V5 tag, which resulted in a similar secretion profile to EscV_wt_-His (Supplementary Figure 2). The single mutants were transformed into Δ*escV*, and their T3SS activity was examined. We observed that mutations G213A and G217A had similar secretion profiles to the Δ*escV* strain transformed with EscV_wt_-V5, while the single mutation G213L completely abolished T3SS activity (Figure 7A). The effect was much milder when the *escV* strain was transformed with the EscV G217L mutant (Figure 7A). To confirm proper expression of the EscV point mutation variants, whole-cell lysates were submitted to western blot analysis using anti-V5 antibody. Comparable protein expression was detected for the WT and the single mutants (Figure 7A). Our results suggest that replacement of the glycine residues of the GxxxG motif found in TMD5 by a large residue (leucine) disrupts the activity of the protein while replacement by a small residue (alanine) does not.

To investigate the effect of the single mutation G213L on the assembly of the T3SS complex, we examined the ability of mutant EscV proteins to properly integrate into the T3SS complex. For this purpose, we grew the EPEC WT and EPEC Δ*escV* strain transformed with EscV_wt_-V5, EscV_G213A_-V5, and EscV_G213L_-V5 under T3SS-inducing conditions. We prepared crude membranes and analyzed them by BN-PAGE and immunoblotting. BN-PAGE analysis showed that the Δ*escV* mutant strain transformed with EscV_wt_-V5 and EscV_G213A_-V5 migrated mainly as a large complex at the top of the gel, while the EscV_G213L_-V5 integration into the complex appeared to be impaired (Figure 7B). To confirm that the altered running pattern of the EscV_G213L_-V5 mutant form was not due to reduced protein expression, the crude membrane extracts were analyzed by SDS-PAGE and immunoblotting using the anti-V5 antibody. We detected a lower expression level of EscV_G213L_-V5 relative to EscV_wt_-V5 and EscV_G213A_-V5, but not to a level that explains the significant reduction in complex formation (Figure 7B). Overall, our results indicate that the GxxxG motif and more specifically the glycine at position 213 are critical for the proper EscV integration into the T3SS complex.

## Discussion

The high conservation of the sequence of EscV TMD6 and the conserved GxxxG motif within TMD5 (Figure 1B), together with the numerous studies regarding TMD-derived oligomerization of membrane complexes (Fink et al., 2012), urged us to examine whether EscV TMDs are involved in protein oligomerization. Results using the isolated ToxR system demonstrated that TMD5 and TMD6 exhibited strong self-oligomerization activities, with activities similar to that of the well-characterized GpA TMD sequence (Figure 2A).

To investigate whether TMD5 and TMD6 sequences are critical for the activity of the full-length protein, we replaced each of these TMDs with an alternative hydrophobic sequence (7L9A). The plasmids encoding TMD5- or TMD6-exchanged EscV versions were transformed into the Δ*escV*-null strain, and their T3SS activity was examined. We found that expression of either EscV-TMD5_ex_-His or EscV-TMD6_ex_-His failed to complement the T3SS activity of the EPEC Δ*escV* strain while the expression of EscV_wt_-His restored T3SS (Figure 4A). Infection of HeLa cells with bacterial strains that express either TMD5- or TMD6-exchanged EscV versions was non-virulent and demonstrated JNK degradation profiles comparable to those of uninfected cells (Figure 4B). Since we observed that the membrane localization of both WT and TMD-exchanged EscVs was not disrupted (Figure 5), we concluded that EscV’s TMD5 and TMD6 are critical not only for proper membrane anchoring but also for T3SS activity and EPEC’s ability to infect host cells as they cannot be replaced by an alternative hydrophobic sequence. Based on the ToxR results, we presume that TMD5 and TMD6 are involved in protein oligomerization although we did not detect complete complex dissociation for T3SS with TMD-exchanged variants (Figure 6). These results suggest that EscV’s TMD5 and TMD6 are not crucial for the global assembly or stability of the T3SS complex but rather that they are involved in promoting the proper TMD–TMD interactions within the complex and their overall orientation to allow passage of T3SS substrates.

To examine the role of the GxxxG motif found within TMD5 on the overall activity of the T3SS, we mutated single glycine residues within the motif, replacing them with either a non-polar small amino acid (alanine) or a non-polar large amino acid (leucine). We found that the original glycine residues could be replaced by alanine residues with no effect on T3SS activity (Figure 7A). These results are in agreement with previous reports suggesting that the GxxxG motif is equivalent to the Small–xxx–Small motif (Lock et al., 2014; Curnow et al., 2020; Wang et al., 2020). In contrast, substitution of leucine for glycine at position 213, but not at 217, abolished T3SS activity (Figure 7A). These results suggested that the two glycine positions do not contribute equally to the activity of the protein and that position 213 is more critical for EscV function within the T3SS complex. Interestingly, while we observed reduced complex formation with the G213L mutation (Figure 7B), we did not observe a similar reduction for EscV TMD5-exchanged (Figure 6), although both had glycine converted to leucine at position 213. These results suggest that TMD–TMD packing is context-dependent and is not dependent on a single residue or motif. Our results are in agreement with previous reports that demonstrated that the GxxxG motif supports TMD interactions within the context of oligo-methionine and oligo-valine sequences, but not within randomized TMDs (Brosig and Langosch, 1998; Unterreitmeier et al., 2007; Langosch and Arkin, 2009).

Expression of EscV_wt_-His within the Δ*escV* null strain unexpectedly resulted in hypersecretion of effectors compared to that seen with WT EPEC (Figure 4A). Interestingly, HA- and V5-tagged EscV expressed from a high copy-number plasmid (pSA10) presented a similar secretion profile, as did expression of HSV-tagged EscV from a low copy-number plasmid (pACYC184, Supplementary Figure 2). A milder phenotype was observed for expression of unlabeled EscV (Supplementary Figure 2). Overall, these results suggested that overexpressing, and to a larger extent, labeling EscV at its C-terminus, regardless of the nature of the tag, interferes with substrate secretion regulation. Our results correlate well with previous studies that indicated that the EscV is involved in substrate secretion regulation through interaction with the “gate-keeper” SepL and several T3SS chaperons (Portaliou et al., 2017; Gaytan et al., 2018). The observation that EscV interacts with SepL via its C-terminal (Portaliou et al., 2017) suggests that labeling EscV at this critical domain disrupts EscV-SepL interaction and therefore induces uncontrolled T3S. This conclusion was further supported by our inability to recapitulate EscV-SepL interaction when EscV was labeled on its C-terminal (data not shown).

Examination of the ability of EPEC Δ*escV* expressing EscV_wt_-His to infect HeLa cells revealed a similar infection capacity as the WT EPEC strain (Figure 4B). This result was unexpected as previous studies revealed that strains with dysregulated T3 substrate secretion (Δ*sepL*, Δ*sepD*, and Δ*escP*) showed reduced infectivity and effector translocation abilities (Deng et al., 2004, 2015; Shaulov et al., 2017). To our knowledge, this is the first example of an EPEC strain that lacks hierarchical substrate secretion regulation but shows similar virulence capabilities to the WT strain. We assume that in contrast to previous strains, the amount of secreted translocators of Δ*escV* that expresses EscV_wt_-His was not reduced, and therefore robust infection was allowed.

In summary, in this work we have shown that TMD5 and TMD6 of EscV are critical for T3SS activity, likely due to their role in TMD–TMD packing within the complex. Further investigation will be required to determine the structural organization within the bacterial inner membrane and to depict the direct interaction partners of EscV within the T3SS complex.

## Data Availability Statement

The raw data supporting the conclusions of this article will be made available by the authors, without undue reservation.

## Author Contributions

BM and NS-M designed the research. BM and SL performed the research. All authors analyzed the data and contributed to the writing and editing of the manuscript.

## Conflict of Interest

The authors declare that the research was conducted in the absence of any commercial or financial relationships that could be construed as a potential conflict of interest.

## Publisher’s Note

All claims expressed in this article are solely those of the authors and do not necessarily represent those of their affiliated organizations, or those of the publisher, the editors and the reviewers. Any product that may be evaluated in this article, or claim that may be made by its manufacturer, is not guaranteed or endorsed by the publisher.

## Footnotes

1
https://www.expasy.org/resources/detail/TMHMM

2
https://www.ebi.ac.uk/Tools/msa/clustalo/
