# Supplementary material for: The Role of the Membrane-Associated Domain of the Export Apparatus Protein, EscV (SctV), in the Activity of the Type III Secretion System
Source: Front Microbiol. 2021 Aug 3;12:719469. doi: 10.3389/fmicb.2021.719469 (PMC8369761; doi:10.3389/fmicb.2021.719469)
Supplement: Supplementary file 1 [file Data_Sheet_1.docx]

**Figure S1**

B7UMA7_ECO27 1 MNKLLNIFK-----KAESYHDLILALFFFMAVMMMIIPLPTVVVDIIIAINISTALLLLM
FLHA_ECOLI 1 MSNLAAMLRLPANLKSTQWQILAGPILILLILSMMVLPLPAFILDLLFTFNIALSIMVLL
Q7DB70_ECO57 1 MNKLLNIFK-----KAESYHDLILALFFFMAVMMMIIPLPTVVVDIIIAINISTALLLLM
A0A2J9SJU1_YERE 1 ML--NQILK-----NARNHPELIILLLMVMIISMLVIPLPTYLVDFLIGLNMVLAILVFM
MXIA_SHIFL 1 MI--QSFLK-----QVSTKPELIILVLMVMIIAMLIIPLPTYLVDFLIGLNIVLAILVFM
A0A0H3NL68_SALT 1 ML--LSLLN-----SARLRPELLILVLMVMIISMFVIPLPTYLVDFLIALNIVLAILVFM
consensus 1 * * . . . * ..*** ..* . *. ... .


B7UMA7_ECO27 56 LSIYIKNPLELTSFPTILLITTLMRLSLSVSTTRLILLHH-----DAGDIIYSFGNFVVG
FLHA_ECOLI 61 VAMFTQRTLEFAAFPTILLFTTLLRLALNVASTRIILMEGHTGAAAAGKVVEAFGHFLVG
Q7DB70_ECO57 56 LSIYIKNPLELTSFPTILLITTLMRLSLSVSTTRLILLHH-----DAGDIIYSFGNFVVG
A0A2J9SJU1_YERE 54 GSFYIDRILSFSSFPSILLITTLFRVALSISTSRLILLEA-----DAGEIITSFGQFVIG
MXIA_SHIFL 54 GSFYIERILSFSTFPSVLLITTLFRLALSISTSRLILVDA-----DAGKIITTFGQFVIG
A0A0H3NL68_SALT 54 GSFYIDRILSFSTFPAVLLITTLFRLALSISTSRLILIEA-----DAGEIIATFGQFVIG
consensus 61 . * ** .** *** *. * . ..*.**. ** .. ** *..*


B7UMA7_ECO27 111 GNIVVGLVIFTIITIVQFMVITKGAERVAEVSARFSLDGMPGKQMSIDGDMRAGVIDPLE
FLHA_ECOLI 121 GNFAIGIVVFVILVIINFMVITKGAGRIAEVGARFVLDGMPGKQMAIDADLNAGLIGEDE
Q7DB70_ECO57 111 GNIVVGLVIFTIITIVQFMVITKGAERVAEVSARFSLDGMPGKQMSIDGDMRAGVIDPLE
A0A2J9SJU1_YERE 109 DSLAVGFVVFSIVTIVQFIVITKGAERVAEVAARFSLDGMPGKQMSIDADLRAGMIDAEM
MXIA_SHIFL 109 DSLAVGFVIFSIVTVVQFIVITKGSERVAEVAARFSLDGMPGKQMSIDADLKAGIIDAAG
A0A0H3NL68_SALT 109 DSLAVGFVVFSIVTVVQFIVITKGSERVAEVAARFSLDGMPGKQMSIDADLKAGIIDADA
consensus 121 .* *.* *. ...*.***** *.*** *** ********* **.*. **.*


B7UMA7_ECO27 171 AKVLRSRVQKESQFYGSMDGAMKFVKGDAIAGIIIVLVNLFGGVLIGMWQFDMPFSEALS
FLHA_ECOLI 181 AKKRRSEVTQEADFYGSMDGASKFVRGDAIAGILIMVINIVGGLLVGVLQHGMSMGHAAE
Q7DB70_ECO57 171 AKVLRSRVQKESQFYGSMDGAMKFVKGDAIAGIIIVLVNLFGGVLIGMWQFDMPFSAALS
A0A2J9SJU1_YERE 169 AKDKRSVLERESQLYGSFDGAMKFIKGDAIANIIIIFVNIIGGLSVGVGQHGMDFSTALS
MXIA_SHIFL 169 AKERRSILERESQLYGSFDGAMKFIKGDAIAGIIIIFVNLIGGISVGMSQHGMSLSGALS
A0A0H3NL68_SALT 169 ARERRSVLERESQLYGSFDGAMKFIKGDAIAGIIIIFVNFIGGISVGMTRHGMDLSSALS
consensus 181 *. ** . * *** *** **..***** *.*. .* **. .*. * *


B7UMA7_ECO27 231 LFSVLSVGDALVAQIPALIISVTAGVVVTRVPGESEKEENLAGDIVQQVSVNSRPFLISA
FLHA_ECOLI 241 SYTLLTIGDGLVAQIPALVISTAAGVIVTRVSTDQD----VGEQMVNQLFSNPSVMLLSA
Q7DB70_ECO57 231 LFSVLSVGDALVAQIPALIISVTAGVVVTRVPGESEKEENLAGDIVQQVSVNSRPFLISA
A0A2J9SJU1_YERE 229 VYTILTVGDGLVSQIPALLIAISAGFIVTRVNGDSD---NMGKSIISQLLRNSFAIVVTA
MXIA_SHIFL 229 TYTILTIGDGLVSQIPALLISISAGFIVTRVNGDSD---NMGRNIMSQIFGNPFVLIVTS
A0A0H3NL68_SALT 229 TYTMLTIGDGLVAQIPALLIAISAGFIVTRVNGDSD---NMGRNIMTQLLNNPFVLVVTA
consensus 241 ...*..**.** *****.* ** .**** . . .. .. *. * ...


B7UMA7_ECO27 291 ALMLVMAIIPGFPTLVFLFLAVCLLGIAWKL--Q-KKRT-------------FGAGNNKD
FLHA_ECOLI 297 AVLGLLGLVPGMPNLVFLLFTAGLLGLAWWI-RG-REQKAPAEPKPVKMAENNTVVEATW
Q7DB70_ECO57 291 ALMLVMAIIPGFPALVFLFLAVCLLGIAWKL--Q-KKRT-------------FGTGNNKD
A0A2J9SJU1_YERE 286 VLALGLGFLPGFPFIVFALLACGLLAYFYFNFWRKNQKKGIADSTSSNIADEFDYAEDS-
MXIA_SHIFL 286 ALALAIGMLPGFPFFVFFLIAVTLTALFYYKKVV-EKEKSLSESDSSGYTGTFDIDNSHD
A0A0H3NL68_SALT 286 ILTISMGTLPGFPLPVFVILSVVLSVLFYFK-FR-EAKRSAAKPKTSKGEQPLSIEEKEG
consensus 301 . .. .** * ** * .


B7UMA7_ECO27 335 AMGADLSNSQ-NISPGAEPLILNLSS-NIYSSDITQQIEVMRWNFFEESGIPLPKIIVNP
FLHA_ECOLI 355 NDVQLEDSLGMEVGYRLIPMVD-----FQQDGELLGRIRSIRKKFAQEMGFLPPVVHIRD
Q7DB70_ECO57 335 AMGADLSNSQ-NISPGAEPLILNLSS-NIYSSDITQQIEVMRWNFFEESGIPLPKIIVNP
A0A2J9SJU1_YERE 345 NALGIINKLD-SVMTETIPLVLLVSHSQVVKLSHSQLSERIRSQFFVEYGIRLPGVIIRE
MXIA_SHIFL 345 SSLAMIENLD-AISSETVPLILLFAENKINANDMEGLIERIRSQFFIDYGVRLPTILYRT
A0A0H3NL68_SALT 344 SSLGLIGDLD-KVSTETVPLILLVPKSRREDLEKAQLAERLRSQFFIDYGVRLPEVLLRD
consensus 361 . *.. .* * . * * .

B7UMA7_ECO27 393 VKN-NDSAIEFLLYQESIYKDTLIDDTVYFEA--------------GHAEISFEFVQEKL
FLHA_ECOLI 410 NMDLQPARYRILMKGVEIGS-----GDAYPGRWLAINPGTAA-GTLPGEATVDPAF----
Q7DB70_ECO57 393 VKN-NDSAIEFLLYQESIYKDTLIDDTVYFEA--------------GHAEISFEFVQEKL
A0A2J9SJU1_YERE 404 GEDLKDNDIVLLLNEVRADQ-----FTIHFDLMRIINYSEDMLSSLDIKPIITAKN----
MXIA_SHIFL 404 SNELKVDDIVLLINEVRADS-----FNIYFDKVCITDENGDI-DALGIPVVSTSYN----
A0A0H3NL68_SALT 403 GEGLDDNSIVLLINEIRVEQ-----FTVYFDLMRVVNYSDEV-VSFGINPTIHQQG----
consensus 421 *.

B7UMA7_ECO27 438 STNSIVYKTNKTNQQLAHLTGMDVYATTNDKITFLLKKLVLSNAKEFIGVQETRYLMDIM
FLHA_ECOLI 460 -GLNAIWIESALKEQAQ-IQGYT-VVEASTVVATHLNHLISQHAAELFGRQEAQQLLDRV
Q7DB70_ECO57 438 STNSIVYKTNKTNQQLAHLTGMDVYATTNDKITFLLKKLVLSNAKEFIGVQETRYLMDIM
A0A2J9SJU1_YERE 455 -DEQYYWVTPEDATKLS-SVGYE-TRSAHDELYKCLSVYLAHNISEYFGIQETKYILDQM
MXIA_SHIFL 454 -ERVISWVDVSYTENLT-NIDAK-IKSAQDEFYHQLSQALLNNINEIFGIQETKNMLDQF
A0A0H3NL68_SALT 453 -SSQYFWVTHEEGEKLR-ELGYV-LRNALDELYHCLAVTLARNVNEYFGIQETKHMLDQL
consensus 481 . * . * * ** ..*


B7UMA7_ECO27 498 ERKYNELVKELQR-QLGLSKIVDILQRLVEENVSIRDLRTIFETLIFWSTKEKDVVILCE
FLHA_ECOLI 517 AQEMPKLTEDLVPGVVTLTTLHKVLQNLLDEKVPIRDMRTILETLAEHAPIQSDPHELTA
Q7DB70_ECO57 498 ERKYNELVKELQR-QLGLSKIVDILQRLVEENVSIRDLRTIFETLIFWSTKEKDVVILCE
A0A2J9SJU1_YERE 512 EMKYPDLLKEVLR-YITIQRIAEVLQRLIQERISVRNMRLVMESLALWAPREKDIIILVE
MXIA_SHIFL 511 ENRYPDLLKEVFR-HVTIQRISEVLQRLLGENISVRNLKLIMESLALWAPREKDVITLVE
A0A0H3NL68_SALT 510 EAKFPDLLKEVLR-HATVQRISEVLQRLLSERVSVRNMKLIMEALALWAPREKDVINLVE
consensus 541 * .. . . .** *. * . .* .. . * * * *


B7UMA7_ECO27 557 YVRIALRRHILGRYSVS-GTLLNVWLIGSDIENELRESIRQTSSGSYLNISPERTEQIIG
FLHA_ECOLI 577 VVRVALGRAITQQWFPGKDEV-HVIGLDTPLERLLLQALQGG-G----GLEPGLADRLLA
Q7DB70_ECO57 557 YVRIALRRHILGRYSVS-GTLLNVWLIGSDIENELRESIRQTSSGSYLNISPERTEQIIG
A0A2J9SJU1_YERE 571 HVRGALARYICHKFAHA-GEI-RAVVVSPILEEKIRNGIRPTAAGTFLNLEPAEAESIID
MXIA_SHIFL 570 HVRASLSRYICSKIAVS-GEI-KVVMLSGYIEDAIRKGIRQTSGGSFLNMDIEVSDEVME
A0A0H3NL68_SALT 569 HIRGAMARYICHKFANG-GEL-RAVMVSAEVEDVIRKGIRQTSGSTFLSLDPEASANLMD
consensus 601 .* . * * . . .* . . . ..


B7UMA7_ECO27 616 F----LKNIMNPTGNGVILTALDIRRYVKKMIEGSFPSVPVLSFQEVGNNIELKVLGTVN
FLHA_ECOLI 631 QTQEALSRQEMLGAPPVLLVNHALRPLLSRFLRRSLPQLVVLSNLELSDNRHIRMTATIG
Q7DB70_ECO57 616 F----LKNIMNPTGNGVILTALDIRRYVKKMIEGSFPSVPVLSFQEVGNNIELKVLGTVN
A0A2J9SJU1_YERE 629 LFKVALDGLNMPIKEIVLLTSVDIRRFIKKFLETSFRDLEVLSYGELTETVSLNVLKTI-
MXIA_SHIFL 628 TLAHALRELRNAKKNFVLLVSVDIRRFVKRLIDNRFKSILVISYAEIDEAYTINVLKTI-
A0A0H3NL68_SALT 627 LITLKLDDLLIAHKDLVLLTSVDVRRFIKKMIEGRFPDLEVLSFGEIADSKSVNVIKTI-
consensus 661 * *.* .* . . . . *.* *. . . *.


B7UMA7_ECO27 672 DFRA
FLHA_ECOLI 691 GK--
Q7DB70_ECO57 672 DFRA
A0A2J9SJU1_YERE ----
MXIA_SHIFL ----
A0A0H3NL68_SALT ----
consensus 721


Figure S1: **Full sequence alignment of the EscV export apparatus protein**. A standard protein BLAST alignment is presented by ClustalW [Larkin, 2007 #1388] for EscV of EPEC T3SS (B7UMA7), FlhA of *E. coli* flagella (P76298), EscV of the E. coli O157:H7 (Q7DB70), YscV of the Yersinia enterocolitica (A0A2J9SJU1), MxiA of the *Shigella* T3SS (P0A1I5), and InvA of the *Salmonella* typhimurium T3SS (A0A0H3NL68).


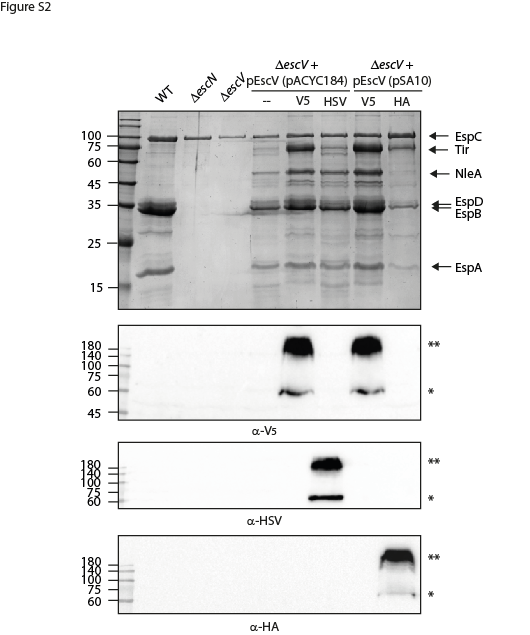


Figure S2: **Labeling EscV at its C-terminal results in enhanced T3SS effector secretion.** Protein secretion profiles of EPEC WT, Δ*escV*, Δ*escN* and EPEC Δ*escV* strain expressing various EscV variants (EscV_wt_, EscV-V5, EscV-HSV, and EscV-HA) grown under T3SS-inducing conditions with 0.1 mM IPTG. The secreted fractions were filtered and protein content was concentrated from the supernatants of bacterial cultures and analyzed by SDS-PAGE and Coomassie blue staining. The T3SS-secreted translocators and effectors EspA, EspB, EspD, NleA and Tir are marked on the right of the gel. Also indicated is the location of EspC, which is not secreted via the T3SS. EscV expression was confirmed in the bacterial pellets by SDS-PAGE and western blot analysis with anti-V5, anti-HSV, and anti-Ha antibodies. Protein bands corresponding to monomeric (*) and multimeric (**) EscV were detected. Numbers on the left are molecular masses in kilodaltons.
